# Toll-like Receptor 9 Mediates Epstein–Barr Virus-Aggravated Inflammation in a Mouse Model of Inflammatory Bowel Disease

**DOI:** 10.3390/biomedicines13071535

**Published:** 2025-06-24

**Authors:** Hassan F. Nour Eddine, Aya M. Kassem, Zahraa Salhab, Nour Sherri, Karen Moghabghab, Zahraa Mohsen, Georges Naim, Sally Mahmoud, Abdo Jurjus, Jana G. Hashash, Elias A. Rahal

**Affiliations:** 1 Department of Experimental Pathology, Immunology, and Microbiology, American University of Beirut, Beirut 1107-2020, Lebanon; hfn08@mail.aub.edu (H.F.N.E.); amk123@mail.aub.edu (A.M.K.); zs83@aub.edu.lb (Z.S.); nas51@mail.aub.edu (N.S.); km78@aub.edu.lb (K.M.); zsm12@mail.aub.edu (Z.M.); gin01@mail.aub.edu (G.N.); sam70@mail.aub.edu (S.M.); 2 Center for Infectious Diseases Research (CIDR), American University of Beirut, Beirut 1107, Lebanon; 3 Department of Anatomy, Cell Biology and Physiological Sciences, American University of Beirut, Beirut 1107-2020, Lebanon; aj00@aub.edu.lb; 4 Division of Gastroenterology and Hepatology, Mayo Clinic, Jacksonville, FL 32224, USA; alhashash.jana@mayo.edu

**Keywords:** autoimmunity, chronic inflammation, Epstein–Barr virus antigens, inflammatory bowel diseases, pro-inflammatory cytokine storm, Toll-like receptor 9

## Abstract

**Background/Objectives**: Inflammatory bowel disease (IBD) is a chronic inflammatory condition encompassing ulcerative colitis (UC) and Crohn’s disease (CD). The role of environmental factors in the pathogenesis of IBD remains elusive. Nevertheless, evidence suggests a pivotal role of viruses, specifically Epstein–Barr virus (EBV), in the progression of IBD through mechanisms such as molecular mimicry and bystander activation. Our previous findings demonstrate EBV DNA’s significant role in exacerbating colitis symptoms and elevating the levels of the pro-autoimmune cytokine interleukin-17A (IL-17A) in an IBD mouse model via toll-like receptor 9 (TLR9). Therefore, we aimed to examine the role of EBV particles in the pathogenesis of IBD, and the potential role of TLR9 inhibition in ameliorating disease outcomes. **Methods**: Three days post colitis induction, EBV particles were intra-rectally injected into female C57BL/6J mice, followed by the intra-peritoneal administration of TLR9 inhibitor. Thereupon, mice were monitored daily and the disease activity index (DAI), colon lengths, and damage scores, as well as the number of cells, double-positive for IL-17A+ and IFN-γ+, and triple-positive for IL-17A+, IFN-γ+, and FOXP3+, were evaluated. **Results**: Our findings revealed a significant role of TLR9 inhibition in mitigating colitis features in an EBV-injected IBD mouse model compared to the control group. **Conclusions**: These results indicate an essential role of TLR9 in initiating immune responses against recurrent EBV reactivation events, which ultimately contributes to inflammation aggravation in IBD patients. Consequently, TLR9 could serve as a potential therapeutic target to alleviate the severe symptoms of IBD in EBV-infected individuals.

## 1. Introduction

EBV, a member of the *Herpesviridae* family, is a human herpesvirus that triggers a wide range of immune responses implicated in the pathogenesis of several autoimmune and inflammatory conditions. Upon infection, EBV can either undergo lytic replication or establish lifelong latency in B lymphocytes. Aging, immunosuppression, stress, or trauma are among the primary factors contributing to the transition of EBV from the latent to the lytic phase [1]. Following reactivation, the virus enters its lytic cycle, releasing viral DNA [2]. Previous research conducted by our team revealed that the intra-peritoneal administration of EBV DNA in mice resulted in elevated levels of the pro-inflammatory cytokine IL-17A throughout the body. This increase in systemic IL-17A levels, in turn, stimulates a pro-autoimmune response, increasing the risk of autoimmune disease development via endosomal Toll-like receptors [3,4]. Recent evidence suggests that EBV may be involved in the pathogenesis of IBD, a chronic and recurring inflammatory disorder with a rising global incidence. The precise etiology of IBD remains elusive, but it is believed to result from the interplay between genetic predisposition, environmental factors, immune dysregulation, and gut microbiome [5]. EBV has been detected in the intestinal mucosa of IBD patients, with studies reporting the presence of EBV-positive cells in the intestinal mucosa of up to 60% of individuals with IBD [6]. Consequently, our team investigated the association between EBV DNA and the aggravation of IBD in *Drosophila melanogaster* and murine models. The findings indicate that EBV DNA elevates the levels of the pro-inflammatory cytokine IL-17A and amplifies intestinal inflammation in a mouse model of IBD via TLRs 3, 7, and 9 [7,8,9]. To further understand the role of viral antigens in the pathogenesis of IBD, we sought to investigate the role of EBV viral particles, rather than the DNA, in exacerbating the severity of intestinal inflammation and disease outcome, and evaluate the therapeutic role of a TLR9 inhibitor in mitigating the severity of IBD in a mouse model.

## 2. Materials and Methods

### 2.1. EBV Induction, Extraction, and Quantification

The P3HR-1 cell line, a type of Burkitt’s lymphoma cell harboring a latent EBV (type 2) strain was purchased from the American Type Culture Collection (ATCC) in Rockville, MD, USA. Cells were cultured in Rosewell Park Memorial Institute (RPMI) 1640 medium (Lonza, Basel, Switzerland) supplemented with 20% fetal bovine serum (Sigma-Aldrich, Darmstadt, Germany) and 1% penicillin–streptomycin (Lonza), and maintained at 70% confluency in a 5% CO_2_ environment at 37 °C. To induce EBV, Phorbol 12-myristate 13-acetate (PMA) (Sigma-Aldrich, Merck KGaA, Darmstadt, Germany) was administered at a concentration of 65 ng/mL for 5 days. Thereupon, the virus-rich supernatant was collected after centrifuging the cells at 800 rpm for 8 min at room temperature. The supernatant was then further processed by centrifugation at 16,000× *g* for 90 min at 4 °C to pellet the virus.

To extract genomic DNA, 100 µL of the viral pellet was mixed with 100 µL of Tris-HCL saturated phenol, vortexed, and centrifuged at 13,000 rpm for 15 min at 4 °C. The upper aqueous layer containing genomic DNA was then combined with 100% cold ethanol and 3 M sodium acetate. The samples were stored overnight at −80 °C to precipitate the viral DNA. The following day, the samples were centrifuged and the DNA pellet was washed twice with 1 mL at 13,000 rpm for 15 min at 4 °C. In the final step, the extracted DNA was resuspended in 30 µL of nuclease-free water.

The number of EBV particles was determined by real-time PCR using the Taq Universal SYBER Green Supermix and the Bio-Rad CFX96TM Real-Time PCR Detection System (Bio-Rad, Berkeley, CA, USA), with specific primers for the EBV-encoded small RNA 2 (*EBER-2*) (Macrogen, Seoul, Republic of Korea) (Table 1). Each PCR tube contained 1 µL of sample DNA in a 10 µL total reaction volume. The thermal cycling conditions included an initial activation at 95 °C for 5 min, followed by 40 cycles of 95 °C for 15 s and 58 °C for 30 s. An EBV DNA standard curve, with a slope of −3.0 to −3.6 and a correlation coefficient of ≥0.98, was used to calculate the number of EBV DNA copies in the P3HR-1 induction preparation.

### 2.2. Acute DSS Colitis Induction and Treatment Administration in C57BL/6J Mice

In accordance with the methodology outlined by Okayasu et al., acute colitis was induced in C57BL/6J mice [10]. Fifty-eight female mice weighing between 15 and 20 g were divided into six groups. The mice, aged between 6 and 8 weeks, were obtained from the American University of Beirut’s Animal Care Facility and handled in compliance with the standards and regulations established by the Institutional Animal Care and Use Committee (IACUC). The mice were supplied with unlimited food and housed in ventilated cages at a temperature of 22–25 °C with a 12-h light–12-h dark cycle. Mice were randomly divided into different groups. All scoring described below was performed independently by two blinded investigators to minimize subjective bias.

Induction of acute colitis was achieved by dissolving DSS (Dextran Sodium Sulfate; MW 40 kDa; Chondrex, Redmond, WA, USA) in drinking water. DSS has been demonstrated to effectively induce colitis through multiple toxic mechanisms including epithelial damage, mucin depletion, and alterations in macrophage functions. Accordingly, the mouse model of DSS-induced colitis is used for the investigation of IBD and extrapolating the results to human conditions [11]. A 1.5% DSS solution was prepared by dissolving 0.75 g of DSS powder in 50 mL of autoclaved distilled water every other day throughout the 7-day period of the experiment.

**Figure 1:**
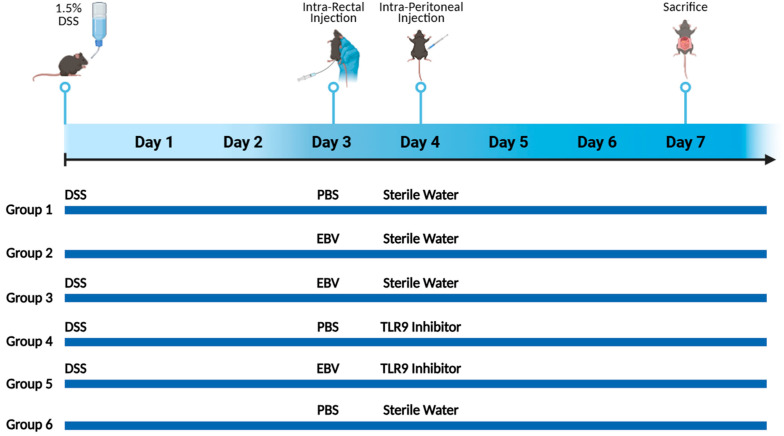
Experimental setup for assessing the effect of EBV particles on the severity of colitis in a DSS-induced IBD mouse model and the role of TLR9 inhibition on damage amelioration. Female C57BL/6J mice in groups 1, 3, 4, and 5 were administered with 1.5% DSS in their drinking water on alternating days for the 7-day experimental period. Mice were assigned to six experimental groups as follows: group 1 (n = 10, DSS), group 2 (n = 10, EBV), group 3 (n = 10, DSS + EBV), group 4 (n = 10, DSS + TLR9 inhibitor), group 5 (n = 10, DSS + EBV + TLR9 inhibitor), and group 6 (n = 8, PBS). Treatments were administered on day 3 via intra-rectal route using either EBV particles or PBS, followed by intraperitoneal injection of TLR9 inhibitor (groups 4 and 5) or sterile water (groups 1, 2, 3, and 6) on day 4.

Mice were randomly assigned into six groups, each consisting of 10 mice (Figure 1). Mice in groups 1, 2, 3, 4, and 5 received an oral administration of freshly prepared DSS in their drinking water, with changes made on alternating days throughout the 7-day experimental period. The DSS dosage was based on optimization studies aimed at identifying the level of colon damage that would permit additional injury from subsequent treatments. On day 3, groups 2, 3, and 5 received an intra-rectal administration of 432 × 10^3^ viral copies in 100 μL of PBS using a 3.5 French catheter. The EBV particle dosage was determined based on data from our systemic EBV studies and previously reported EBV levels in colonic tissues. Groups 1, 4, and 6 served as controls, receiving only 100 μL of PBS. Prior to every intra-rectal injection, a comprehensive abdominal massage was performed to expel any potential fecal matter, and ensure the retention of the viral content introduced through rectal gavage. On the fourth day, groups 4 and 5 were intraperitoneally injected with 56 μg of the TLR 9 antagonist ODN2088 (Macrogen, Seoul, Republic of Korea) (Table 2), dissolved in 100 μL of nuclease-free water. On day 7, all mice were euthanized under sevoflurane anesthesia via midline laparotomy, followed by cardiac puncture for tissue and blood collection. No deaths occurred among the treated mouse groups during the monitoring period.

### 2.3. Mouse Monitoring and Assessment

The severity of colitis in each group was assessed daily starting from Day 0. The assessment involved recording each mouse’s body weight, stool consistency, and blood in stool. In cases where gross bleeding was not visible, an occult blood test was performed. These observations were used to calculate the DAI according to the method described by Cooper et al. [12] (Table 3). Following sacrifice, the colons were excised and their lengths were measured, with a shorter colon length indicating enhanced colitis severity [13]. Additionally, colon lumens were rinsed with cold phosphate-buffered saline (PBS), and sections from the distal end were excised and fixed in 10% formaldehyde overnight prior to paraffin embedding. Hematoxylin and eosin (H&E) staining was performed on five-micrometer sections from the distal colon previously deparaffinized using xylene and rehydrated through a graded series of ethanol washes (100%, 95%, and 70%). The extent of crypt damage and inflammation was then assessed to grade histological damage, as detailed in Table 4 [14]. The cross-sections were graded based on inflammation severity (immune cell infiltration), inflammation extent (inflammation limited to the mucosa or advanced transmurally through the sub-mucosa towards the muscularis layer), and crypt damage (basal damage to the crypt/complete crypt loss). These grades were summed to determine the histological damage score.

### 2.4. Assessing the Number of Single, Double, and Triple Positive T Cells in Colon Tissues

Immunofluorescence was performed to detect T cells expressing IL-17A+, IFN- γ+, or FOXP3+, as well as double-positive (IL-17A+, IFN-γ+) and triple-positive (IL-17A+, IFN- γ+, FOXP3+) populations in colon tissues. Initially, the unstained tissue sections were heated at 55 °C for 40 min, then immersed in xylene twice for 10 min per immersion for deparaffinization. Then, the slides were rehydrated through a series of decreasing ethanol concentrations (100%, 95%, and 75%) for five minutes each, followed by a double rinse in distilled water for five minutes. Afterward, the slides were immersed in citrate buffer (pH = 6) for 90 min in a water bath maintained at 60 °C, enhancing the efficiency of antigen retrieval. The preparation of the citrate buffer was carried out using 0.1 M tri-sodium citrate dihydrate and 0.1 M citric acid (18 mL of citric acid, 82 mL of tri-sodium citrate dehydrate, 900 mL distilled water). Subsequently, the slides were cooled for 30 min and washed twice with distilled water for 5 min. The sections were permeabilized for 5 min using 0.3% Triton X-100 in 1× PBS to allow antibody penetration, then washed with distilled water once for another 5 min. For the blocking step, slides were incubated in a blocking buffer (composed of 15% FBS in 1× PBS) for 30 min at room temperature and then washed two times with distilled water. An antibody dilution buffer was prepared using 1× PBS, 15% FBS, and 0.3% Triton X-100. The slides were incubated overnight with three fluorochrome-linked primary antibodies: Brilliant Violet 605 anti-mouse IL-17A (1:500), Pacific Blue 405 anti-mouse IFN-γ (1:500), and Alexa Fluor 488 anti-mouse FOXP3 (1:500) (Biolegend, CA, USA). The next day, the slides underwent a three-time wash with 1× PBS to remove unbound antibodies, and then were covered with a mounting solution (80% glycerol, 223 mM 1,4-diazabicyclo (2.2.2) octane (DABCO), and 4 mM Tris-HCl) and a cover slip, and stored at 4 °C. Slides were observed using a Leica DM4B Upright Fluorescence Microscope using the Leica 2.0.0.14332 software. The quantification of single, double, and triple-positive cells per unit area was determined using ImageJ v1.54i (National Institutes of Health, Rasband, WS, USA) manually, by 2 independent operators, and expressed as log foci per inch^2^.

### 2.5. Statistical Analysis

Statistical analyses were performed using GraphPad Prism 8.4.3 software. Power analysis was performed using the AEEC Animal Experimentation Sample Size Calculator (http://www.lasec.cuhk.edu.hk/sample-size-calculation.html, accessed on 10 June 2025). DAI and histological scores were compared using the Mann–Whitney U test. Normally distributed continuous variables were analyzed by unpaired t-test, with normality confirmed by Shapiro–Wilk testing. Potential outliers were identified using Grubbs’ test. A *p*-value < 0.05 was considered statistically significant.

## 3. Results

### 3.1. EBV Enhances the Disease Activity Index (DAI) in the Dextran Sodium Sulfate (DSS) Mouse Colitis Model in a TLR9-Dependent Manner

**Figure 2:**
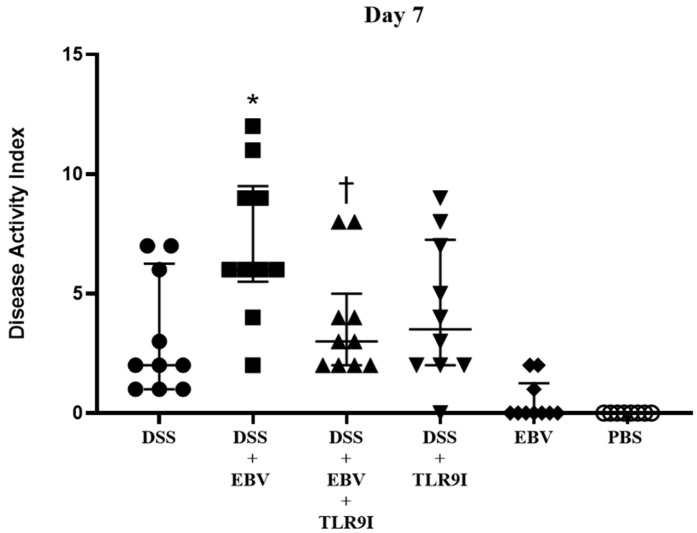
Disease activity index scores on day 7 assessing the effect of EBV particles on the severity of colitis in a DSS-induced IBD mouse model and the role of TLR9 inhibition on damage amelioration. Mice were assigned to six experimental groups as follows: group 1 (n = 10, DSS), group 2 (n = 10, DSS + EBV), group 3 (n = 10, DSS + EBV + TLR9 inhibitor), group 4 (n = 10, DSS + TLR9 inhibitor), group 5 (n = 10, EBV), and group 6 (n = 8, PBS). Treatments were administered on day 3 via intra-rectal route using either EBV particles or PBS, followed by intraperitoneal injection of TLR9 inhibitor (groups 4 and 5) or sterile water (groups 1, 2, 3, and 6) on day 4. * *p*-value < 0.05, compared to DSS group; † *p*-value < 0.05, compared to DSS + EBV.

The effect of EBV and TLR9 inhibition on the severity of colitis in an IBD mouse model was evaluated using the DAI (Figure 2). A progressive rise in DAI scores was observed in all groups administered DSS. By the seventh day, the group receiving DSS and EBV particles had an average DAI score of 7.1, which was significantly higher (*p* = 0.0153) compared to the DSS-administered group with an average score of 3.2. On the other hand, the group administered DSS along with EBV and TLR9 inhibitor exhibited a significant reduction (*p* = 0.0163) in the DAI score, averaging at 3.8, in comparison to the DSS-administered EBV-treated group, averaging at 7.1. Moreover, the administration of TLR9 inhibitor to the DSS-treated group showed no difference in the average DAI score compared to the DSS-treated one (*p* = 0.3355). As for the groups administered EBV or PBS, the average DAI scores were 0.5 and 0, respectively, suggesting no observable impact.

### 3.2. EBV Administration Decreases Colon Lengths in the Dextran Sodium Sulfate (DSS) Mouse Colitis Model in a TLR9-Dependent Manner

**Figure 3:**
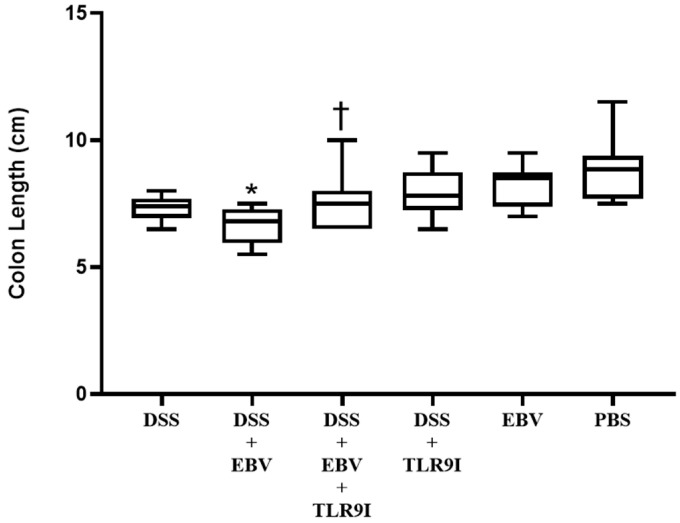
Colon lengths on day 7 assessing the effect of EBV particles on the severity of colitis in a DSS-induced IBD mouse model and the role of TLR9 inhibition on damage amelioration. Mice were assigned to six experimental groups as follows: group 1 (n = 10, DSS), group 2 (n = 10, DSS + EBV), group 3 (n = 10, DSS + EBV + TLR9 inhibitor), group 4 (n = 10, DSS + TLR9 inhibitor), group 5 (n = 10, EBV), and group 6 (n = 8, PBS). Treatments were administered on day 3 via intra-rectal route using either EBV particles or PBS, followed by intraperitoneal injection of TLR9 inhibitor (groups 4 and 5) or sterile water (groups 1, 2, 3, and 6) on day 4. * *p*-value < 0.05, compared to DSS group; † *p*-value < 0.05, compared to DSS + EBV.

Consistent with the DAI scores, the colon length was significantly shorter in the group receiving DSS and EBV (*p* = 0.0296) in comparison to the DSS-treated controls. The former group had an average colon length of 6.66 cm, while the latter demonstrated an average of 7.31 cm. The group intraperitoneally injected with TLR9 inhibitor alongside the administration of DSS and EBV, experienced a significant augmentation in colon length compared to that treated with EBV and DSS (*p* = 0.0423), with an average length of 7.53 cm. Interestingly, the intraperitoneal treatment with TLR9 inhibitor in conjugation with DSS resulted in an increase in colon length, with an average of 7.96 cm in comparison to 7.31 cm in the control group. This increase, however, was not significant (*p* = 0.0979). The groups receiving EBV or PBS only had an average colon length of 8.25 and 8.85 cm, respectively. The colon length results are presented in Figure 3.

### 3.3. EBV Administration Increases the Histological Damage Score in the Dextran Sodium Sulfate (DSS) Mouse Colitis Model in a TLR9-Dependent Manner

**Figure 4:**
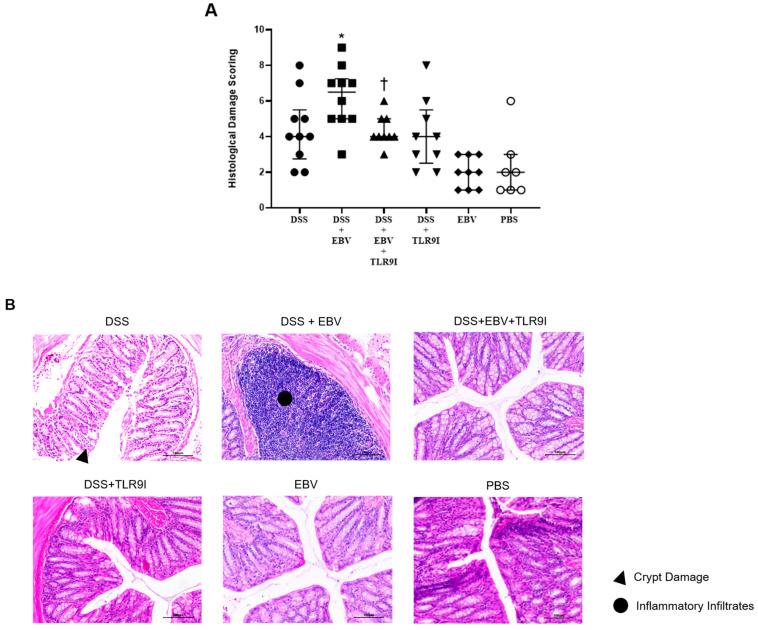
(**A**,**B**) Histological damage scores in colon tissue and H&E-stained sections assessing the effect of EBV particles on the severity of colitis in a DSS-induced IBD mouse model and the role of TLR9 inhibition on damage amelioration (Scale bar = 100 µm). Mice were assigned to six experimental groups as follows: group 1 (n = 10, DSS), group 2 (n = 10, DSS + EBV), group 3 (n = 9, DSS + EBV + TLR9 inhibitor), group 4 (n = 9, DSS + TLR9 inhibitor), group 5 (n = 9, EBV), and group 6 (n = 7, PBS). Treatments were administered on day 3 via intra-rectal route using either EBV particles or PBS, followed by intraperitoneal injection of TLR9 inhibitor (groups 4 and 5) or sterile water (groups 1, 2, 3, and 6) on day 4. * *p*-value < 0.05, compared to DSS group; † *p*-value < 0.05, compared to DSS + EBV.

Following sacrifice, H&E-stained sections of the colon were prepared to assess the histological damage induced by the different treatments on the tissues. In line with previous results, the histological damage score was markedly enhanced (*p* = 0.0412) in the group receiving EBV and DSS (average score = 6.2) relative to the DSS group (average score = 4.4). Notably, the administration of TLR9 inhibitor alongside DSS and EBV significantly attenuated (*p* = 0.0110) the average damage score from 6.2 in the DSS-induced EBV-treated group to 4.33. Moreover, the group receiving DSS and TLR9 inhibitor demonstrated a damage score of 4.11. This reduction was not significant compared to the DSS group (*p* = 0.7289). Histological analysis revealed similar low-level damage in the EBV and PBS treatment groups, with average scores of 2 and 2.8, respectively, indicating neither intervention significantly altered disease progression. The histological damage scoring results are presented in Figure 4 below.

### 3.4. EBV Administration Increases Single Positive IL-17A+, IFN-γ+, and FOXP3+ Cells, Double Positive IL-17A+/IFN-γ+, and Triple Positive IL-17A+/IFN-γ+/FOXP3+ Counts in the Dextran Sodium Sulfate (DSS) Mouse Colitis Model in a TLR9-Dependent Manner

**Figure 5:**
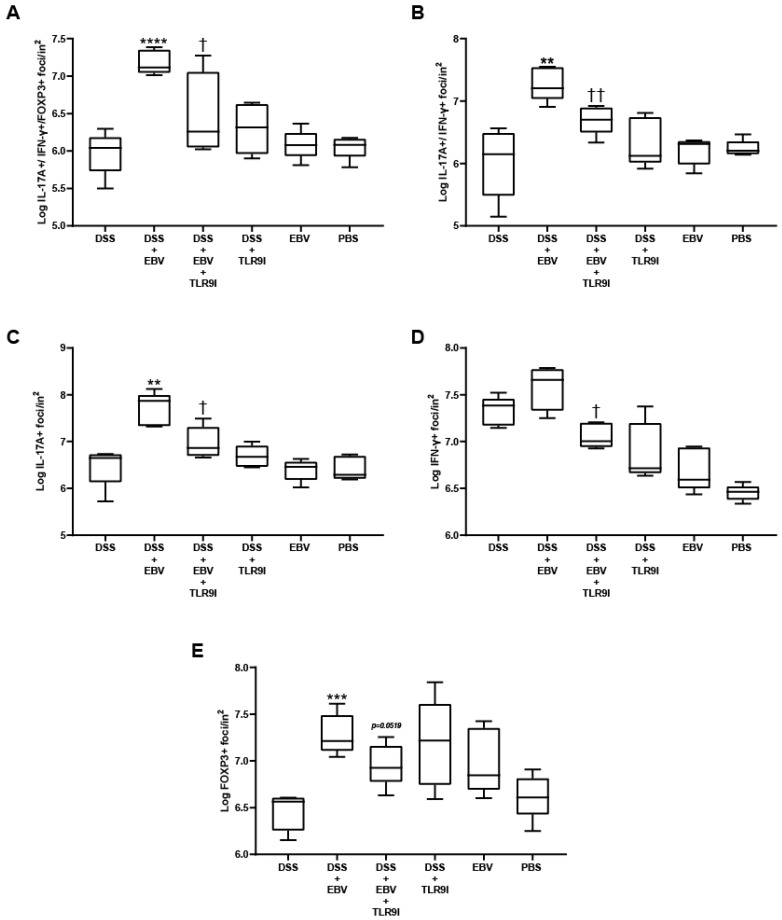
(**A**–**E**) Immunofluorescence staining assessing the effect of EBV particles on the counts of single positive IL-17A+, IFN-γ+, and FOXP3+ cells, double positive IL-17A+/IFN-γ+, and triple positive IL-17A+/IFN-γ+/FOXP3+ cells in a DSS-induced IBD mouse model and the role of TLR9 inhibition on damage amelioration. All mouse groups (n = 5) were intra-rectally administered with EBV particles (groups 2, 3, and 5) or PBS (groups 1, 4, and 6) on day 3 followed by the intraperitoneal injection of TLR9 inhibitor (groups 4 and 5) or sterile water (groups 1, 2, 3, and 6) on day 4. ** *p*-value < 0.01, compared to DSS group; *** *p*-value < 0.001, compared to DSS group; **** *p*-value < 0.0001, compared to DSS group; † *p*-value < 0.05, compared to DSS + EBV; †† *p*-value < 0.01, compared to DSS + EBV.

Immunofluorescent labeling of inflammatory markers was performed on colon cross-sections of control and experimental mouse groups as shown in Figure 5. Mice administered DSS and EBV had the highest number of the pathogenicity-associated double positive IL-17A+/IFN-γ+ foci, with 7.27 log(foci/in^2^). This increase was significant (*p* = 0.0021) compared to the DSS group with 6.01 log(foci/in^2^). When the TLR9 inhibitor was injected alongside DSS and EBV, results revealed a significant reduction (*p* = 0.0069) in double positive foci to 6.69 log(foci/in^2^). Furthermore, the group receiving DSS and TLR9 inhibitor showed a higher average of 6.32 log(foci/in^2^) compared to the DSS group; however, the difference was not statistically significant (*p* = 0.3468). The EBV- and PBS-treated groups had averages of 6.20 and 6.24 log(foci/in^2^), respectively.

Similarly, the group receiving DSS and EBV had significantly elevated IL-17A+/IFN-γ+/FOXP3+ triple positive foci in comparison with the DSS group (*p* < 0.0001), with counts rising from 5.97 to 7.18 log(foci/in^2^). TLR9 inhibition decreased (*p* = 0.0266) the pathogenic cell counts to 6.49 log(foci/in^2^) in group 3. Furthermore, our data revealed a non-significant elevation (*p* = 0.1445) in triple positive counts to 6.29 log(foci/in^2^) in the DSS-induced TLR9 inhibitor-treated group compared to the DSS-induced group (*p* = 0.1445). As for the groups receiving EBV or PBS, the average counts were 6.08 and 6.05 log(foci/in^2^), respectively.

**Figure 6:**
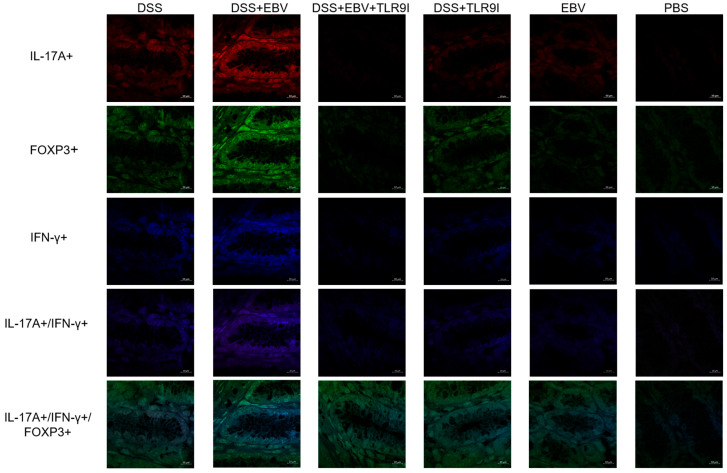
Confocal images assessing the effect of EBV particles on the counts of single positive IL-17A+, IFN-γ+, and FOXP3+ cells, double positive IL-17A+/IFN-γ+, and triple positive IL-17A+/IFN-γ+/FOXP3+ cells in a DSS-induced IBD mouse model and the role of TLR9 inhibition on damage amelioration (Scale bar = 10 µm). All mouse groups (n = 5) were intra-rectally administered with EBV particles (groups 2, 3, and 5) or PBS (groups 1, 4, and 6) on day 3 followed by the intraperitoneal injection of TLR9 inhibitor (groups 4 and 5) or sterile water (groups 1, 2, 3, and 6) on day 4.

In the DSS-induced EBV-treated group, a marked increase was observed in the number of cells single-positive for IL-17A+, IFN-γ+, or FOXP3+ compared to the DSS group. Specifically, IL-17A+ cell counts demonstrated a statistically significant increase (*p* = 0.0011) from 6.47 to 7.70 log(foci/in^2^). Similarly, FOXP3+ cell counts also showed a significant elevation (*p* = 0.0002), rising from 6.45 to 7.28 log(foci/in^2^). In contrast, while IFN-γ+ cell counts increased from 7.32 to 7.57 log(foci/in^2^), this change did not reach statistical significance (*p* = 0.0874). On the other hand, the TLR9 inhibition reduced the counts of single-positive cells. IL-17A+ cell counts significantly decreased to 6.97 (*p* = 0.0108), IFN-γ+ cell counts declined to 7.05 log(foci/in^2^) (*p* = 0.0027), and FOXP3+ cell counts were reduced to 6.96 log(foci/in^2^), although the reduction in FOXP3+ cells reached only borderline statistical significance (*p* = 0.0519). Confocal images assessing the number of single, double, and triple positive pathogenic cells in colon tissue across experimental groups are represented in Figure 6.

## 4. Discussion

In 1975, a report by the International Agency for Research on Cancer (IARC) states that more than 95% of the population shows serum seropositivity for EBV [15]. A 2020 sero-epidemiological study using serum samples confirmed that EBV seroprevalence remains consistently high, exceeding 95% among individuals aged 21 to 25. The findings also indicate a notable upward trend in EBV seroprevalence worldwide [16]. EBV has been linked to multiple non-malignant conditions such as infectious mononucleosis (IM), as well as malignant conditions including Burkitt’s lymphoma (BL), Hodgkins’s lymphoma (HL), and nasopharyngeal carcinoma (NPC). Due to immune system alterations triggered by EBV proteins and the virus’s ability to persist in B cells, EBV serves as a major risk factor for autoimmune and inflammatory diseases such as systemic lupus erythematosus (SLE), rheumatoid arthritis (RA), and IBD [17]. It has been demonstrated that there exists a high-affinity molecular mimicry between the Epstein–Barr nuclear antigen 1 (EBNA-1) and the central nervous system protein glial cell adhesion molecule (Glial CAM), with structural and functional evidence for its relevance. These findings underscore the role of EBV as a contributing factor to multiple sclerosis (MS) through mechanisms involving immune cross-reactivity [18].

This study aims to understand the mechanistic role of EBV in IBD as it is widely recognized to exacerbate inflammation in patients with autoimmune and inflammatory conditions. EBV was shown to increase the levels of IL-18, a pro-inflammatory cytokine, in EBV-associated diseases [19]. Moreover, EBV-positive Sjögren’s Syndrome patients had higher IL-21-producing T cells compared to controls [20], and elevated blood EBV DNA levels correlated with increased levels of IL-17A in rheumatoid arthritis patients but not in controls. These findings highlight the relevance of developing management approaches to EBV [21].

In an attempt to assess the immune-stimulatory properties of EBV and its potential link to autoimmunity, our lab conducted extensive studies on mouse models. We primarily focused on EBV DNA, as it is known to be shed during viral reactivation and possesses immunostimulatory properties. EBV DNA is hypothesized to contribute to the onset of autoimmune and inflammatory diseases, or exacerbate pre-existing conditions. Our preliminary data indicated that EBV DNA elevates the systemic levels of the pro-inflammatory cytokine IL-17A, a marker that is highly correlated with autoimmunity [4]. Because EBV DNA is rich in CpG motifs, it is effectively recognized by TLR9, a member of the pattern recognition receptor (PRR) family that specifically detects dsDNA. Subsequent research findings validated that EBV DNA enhances the expression of the endosomal TLRs 3, 7, and 9, leading to enhanced levels of the pro-autoimmune cytokine IL-17A. The inhibition of these receptors significantly reduced IL-17A levels, emphasizing their role in mediating immune responses triggered by EBV [3,21].

Although the excessive production of IL-17A promotes chronic inflammation in IBD, reports highlight the pivotal protective role of this cytokine in IBD patients. Blocking IL-17A using antibodies in a DSS-induced IBD mouse model exacerbated intestinal inflammation. This aggravation mainly stemmed from compromised integrity of the intestinal epithelial barrier, increased intestinal permeability, diminished expression of antimicrobial peptides, and decreased aggregation of neutrophils [22,23]. Targeting IL-17A may not only impact inflammatory immune cells but also disturb the balance of IL-17A-producing cells responsible for maintaining homeostasis, potentially exacerbating the condition.

A significant correlation has been established between EBV prevalence and the clinical disease activities of IBD, averaging a staggering 75% presence of EBV-positive cells within the mucosa of severe cases of IBD [5]. Previous lab findings demonstrated exacerbated gut inflammation in the presence of EBV DNA in both *Drosophila melanogaster* and mouse models [7,8]. Additionally, the usage of a TLR9 antagonist managed to alleviate intestinal inflammation in a mouse model of IBD [9]. Therefore, we sought to investigate the role of EBV particles, irrespective of viral replication, on colitis severity and the involvement of TLR9 inhibition in ameliorating intestinal inflammation in an IBD mouse model.

To address this, colitis was induced in female C57BL/6 mice aged 6–8 weeks through the administration of DSS in their drinking water. This model of gut inflammation closely resembles human UC, with symptoms including weight loss, diarrhea, fecal occult blood, colon shortening, and mucosal ulceration [24,25]. A concentration of 1.5% DSS was utilized based on previous studies indicating moderately severe clinical symptoms and colonic shrinkage in IBD models [8]. For optimizing the viral particle dosage to be implemented in our experimental model, six groups of five female C57BL/6J mice, aged 6–8 weeks were utilized. The groups received increasing EBV copy numbers of 144 × 10^3^, 288 × 10^3^, 432 × 10^3^, 576 × 10^3^, and 720 × 10^3^. We opted for 432 × 10^3^ EBV particles as this group showed significantly higher DAI scores compared to the DSS-treated group with no signs of hyperinflammation. On the other hand, earlier laboratory data demonstrated that the intra-peritoneal administration of 56 µg of ODN2088 (TLR9 inhibitor) in 100 µL of sterile water is sufficient to reduce IL-17A serum levels in response to the systemic administration of EBV DNA in mice. Hence, this concentration of TLR9 antagonist was implemented. The effect of EBV in a mouse model of IBD and the effect of TLR9 inhibition on EBV-exacerbated colitis was evaluated based on trends in the DAI, macroscopic assessments of the colon, histological damage scoring of the H&E-stained colon cross sections, and immunofluorescence staining of pro-inflammatory markers in colon tissue.

Upon assessing the DAI on day 7, the group intra-rectally administered EBV alongside DSS exhibited a significant increase in the DAI score in comparison to the DSS-induced controls, indicating that EBV exacerbates the clinical manifestations of colitis in a mouse model of IBD. Conversely, the group receiving an intra-peritoneal injection of TLR9 inhibitor in addition to EBV and DSS displayed a significantly lower DAI score by the seventh day compared to the former group. Hence, the data indicates that the worsened clinical manifestations triggered by EBV are mediated by TLR9.

Consistently, the group administered DSS and EBV demonstrated significantly shorter colons on sacrifice day in comparison to the group solely receiving DSS. On the other hand, TLR9 inhibition significantly prevented this shortening in the group receiving DSS, EBV, and the TLR9 inhibitor. This suggests that the EBV-stimulated inflammation further contributes to colon shortening in a DSS-induced mouse model of IBD in a TLR9-dependent manner.

As for the histological damage scores in the colon, it was significantly higher in mice treated with DSS and EBV compared to the DSS-treated group, further demonstrating the worsening and damaging role of EBV. Interestingly, the TLR9 antagonist exhibited significant efficacy in lowering the DSS-EBV-induced damage. Altogether, our findings provide a more comprehensive depiction of how EBV exacerbates intestinal inflammation within a mouse model of IBD and the role of TLR9 inhibition in mitigating the clinical severity of the disease, as evidenced by lower DAI and damage scores, as well as longer colon lengths.

Under normal circumstances, IL-17A is produced in response to extracellular bacteria and fungi, recruiting neutrophils to the affected site. However, certain infections trigger excessive production of IL-17A, thereby promoting the development or aggravation of inflammatory diseases such as IBD. Upon intestinal inflammation, Th17 cells infiltrate the gastrointestinal mucosa of CD and UC patients, stimulating an elevation in expression levels of the pro-autoimmune cytokine IL-17A [26]. Furthermore, a series of studies in CD45RB models have established a link between the onset of colitis and IFN-γ production by Th1 cell lineage [27]. The role of IFN-γ in IBD has been established in other studies as well [28]. There have been findings indicating a potential interaction between IL-17A and IFN-γ, governing inflammation within the gastrointestinal tract. For instance, IFN- γ- producing TH17 cells have been detected in the gut of subjects with CD [29]. Our data revealed a significant increase in the number of cells double-positive for IL-17A+ and IFN-γ+ in DSS-induced EBV-treated mice. These findings are consistent with prior research demonstrating elevated levels of IL-17A and IFN-γ-producing cells in autoimmune diseases such as MS. This highlights the functional plasticity of T cells, where IL-17A-producing cells adopt a distinct and highly pathogenic phenotype upon upregulating the T-box transcription factor (T-bet), leading to the production of IFN-γ, and untimely resulting in disease progression. On the other hand, the Forkhead box protein 3 (FOXP3), an essential transcription factor expressed by regulatory T cells (Tregs), is involved in the regulation of their development and function. Tregs are vital for maintaining immune system homeostasis and ensuring that immune responses are appropriately controlled to prevent excessive reactions. However, increasing evidence highlights defects in the suppressive role of Tregs in several autoimmune diseases. In our study, we observed a marked increase in the number of triple-positive cells co-expressing IL-17A+, IFN-γ+, and FOXP3+ in this group. These cells likely originate from regulatory T cells that, under inflammatory conditions, lose their suppressive function and acquire pro-inflammatory properties. This transition results in a hybrid state, where Tregs co-express ROR-γt, T-bet, and FOXP3 [30]. Notably, the retention of FOXP3 in these cells appears insufficient to maintain immune regulation, underscoring the profound impact of the inflammatory environment on Treg stability and function. These findings provide further evidence of the dynamic interplay between regulatory and effector immune responses in chronic inflammation and highlight the potential role of T cell plasticity in disease progression. Nevertheless, TLR9 inhibition successfully lessened the count of these pathogenic cells in the dominant pro-inflammatory environment driven by EBV, emphasizing the critical role of these receptors in disease progression.

## 5. Conclusions

Our study indicates that EBV aggravates colitis symptoms in an IBD mouse model through TLR9. The activation of TLR9 by EBV is likely mediated through the unmethylated CpG sequences present in EBV DNA, which serve as the ligand and activator of TLR9. This interaction triggers signaling pathways that promote the production of inflammatory cytokines associated with autoimmunity, such as IL-17A and IFN-γ. Therefore, leveraging TLR9 inhibitors in managing IBD symptoms provides a possible avenue for therapeutic or preventive intervention in EBV-infected individuals. While this study offers significant insights, it is important to acknowledge certain limitations. The DSS-induced colitis model demonstrates anatomical, clinical, and histological similarities with human UC, and thus more accurately reflects UC than CD. Furthermore, the mouse model used lacks the CD21 receptor essential for EBV entry and infection of B cells. Hence, the observed effects are attributed to the immune response induced by the distinct EBV antigens, rather than the active viral replication. Humanized mice expressing the CD21 receptor could serve as an appropriate model for the study of EBV infection and replication in an environment relevant to human physiology. This would provide enhanced clarity and a comprehensive understanding of the immune response triggered by EBV and its contribution to the development and pathogenesis of EBV-associated diseases including IBD. Additionally, the study utilized an acute model of DSS, which might not adequately represent the chronic inflammation characteristic of IBD.

Further research concerning the evaluation of different dosage regimens and delivery approaches for TLR9 inhibitors is essential to enhance its effectiveness. Moreover, assessing the role of other PRRs in response to EBV, specifically TLRs 3 and 7, which were previously reported to be involved in the EBV DNA-mediated elevation of IL-17A, could provide more insights into the mechanism by which EBV contributes to inflammation. In conclusion, a better understanding of the role of viral replication and reactivation in the pathogenesis of inflammatory diseases is of great importance for the management of IBD manifestations in EBV-infected individuals.

## Figures and Tables

**Table 1:** Primer sequence and annealing temperature for the *EBER-2* gene.

Gene	Primer Sequence	Annealing Temperature
*EBER-2*	F: 5′-CCCTAGTGGTTTCGGACACA-3′	58 °C
	R: 5′-ACTTGCAAATGCTCTAGGCG-3′	58 °C

**Table 2:** Toll-like receptor 9 inhibitor: nucleotide sequence and dosage details.

TLR9 Inhibitor	Sequence	Dosage Concentration
ODN2088	5′-TCCTGGCGGGGAAGT-3′	56 μg in 100 μL

**Table 3:** Disease activity index (DAI) scoring system for the evaluation of colitis severity in mice.

	Clinical Parameters		
Score	Weight Loss (%)	Stool Consistency	Blood in Feces
0	None	Normal	None
1	1–5	-	-
2	6–10	Loose Stools	Occult Bleeding
3	11–15	-	-
4	>15	Diarrhea	Gross Bleeding

**Table 4:** Scoring criteria for histological damage in colon tissue.

Scoring Parameters	Grade	Grading Criteria
Inflammation Severity	0	None
	1	Mild
	2	Moderate
	3	Severe
Inflammation Extent	0	None
	1	Mucosa
	2	Submucosa
	3	Transmural
Crypt Damage	0	None
	1	1/3 Basal Damage
	2	2/3 Basal Damage
	3	Crypt Damage with Intact Surface Epithelium
	4	Crypt Damage with Complete Loss of Epithelial Surface

## Data Availability

Data are available upon request submitted to the corresponding author.

## Abbreviations

The following abbreviations are used in this manuscript:

IBD	Inflammatory Bowel Diseases
UC	Ulcerative Colitis
CD	Crohn’s Disease
EBV	Epstein–Barr Virus
IL-17A	Interleukin 17A
TLR9	Toll-like Receptor 9
DAI	Disease Activity Index
IFN-γ	Interferon γ
FOXP3	Forkhead Box Protein 3
DSS	Dextran Sodium Sulfate
PBS	Phosphate Buffered Saline
IM	Infectious Mononucleosis
BL	Burkitt’s Lymphoma
HL	Hodgkins Lymphoma
NPC	Nasopharyngeal Carcinoma
SLE	Systemic Lupus Erythematosus
RA	Rheumatoid Arthritis
EBNA-1	Epstein-Barr Nuclear Antigen 1
Glial CAM	Glial Cell Adhesion Molecule
MS	Multiple Sclerosis
PRR	Pattern Recognition Receptor
T bet	T-box Transcription Factor
Treg	Regulatory T cell
RORγt	Retinoic Acid-Related Orphan Receptor γ T
